# Long-term smoking increases the need for acute care among asthma patients: a case control study

**DOI:** 10.1186/1471-2466-14-119

**Published:** 2014-07-16

**Authors:** Paula Kauppi, Henna Kupiainen, Ari Lindqvist, Tari Haahtela, Tarja Laitinen

**Affiliations:** 1 Skin and Allergy Hospital, Helsinki University Central Hospital, PO Box 160, FI-00029 HUS Helsinki, Finland; 2 Research Unit of Pulmonary Diseases and Division of Pulmonology, Helsinki University Central Hospital, PO Box 340, FI-00029 HUS Helsinki, Finland; 3 Department of Pulmonary Diseases and Clinical Allergology, Turku University Hospital and University of Turku, PO BOX 52, FI-20521 Turku, Finland

**Keywords:** Obstructive lung disease, Emergency visits, Hospitalizations, Smoking cessation

## Abstract

**Background:**

To examine risk factors for asthma patients’ emergency room (ER) visits in a well organized asthma care setting.

**Methods:**

A random sample of 344 asthma patients from a Pulmonary Clinic of a University Hospital were followed through medical records from 1995 to 2006. All the ER visits due to dyspnea, respiratory infections, chest pain, and discomfort were evaluated.

**Results:**

The mean age of the study population was 56 years (SD 13 years), 72% being women. 117 (34%) of the patients had had at least one ER visit during the follow-up (mean 0.5 emergency visits per patient year, range 0–7). Asthma exacerbation, lower and upper respiratory infections accounted for the 71% of the ER visits and 77% of the hospitalizations. The patients with ER visits were older, had suffered longer from asthma and more frequently from chronic sinusitis, were more often ex- or current smokers, and had lower lung function parameters compared to the patients without emergency visits. Previous (HR 1.9, CI 1.3-3.1) and current smoking (HR 3.6, CI 1.6-8.2), poor self-reported health related quality of life (HRQoL) (HR 2.5, CI 1.5-4), and poor lung function (FEV1 < 65%, HR 2.2, CI 1.3-3.7) remained independent risk factors for ER visits after adjustment for age and gender.

**Conclusions:**

Asthma patients who are or have been long-term smokers are more likely to require ER care compared to never smokers.

## Background

Modern pharmaceuticals such as inhaled steroids, long-acting β2-agonists, their combinations, and leukotriene antagonists have allowed high quality asthma management [1,2]. The present concept of asthma management is to intensify the treatment until full control has been achieved. In Finland, asthma management has developed not only because of efficient medication but also due to a nationwide educational programme during years 1994–2004 [3]. The program was focused to improve the patients’ self-management capabilities by implementation of a self-management plan, and to update the referral and treatment networks in primary health care [3]. Improved care was proven by several indicators, such as decrease in mortality, disability pensions, and hospital days, and other savings in health care. Better control of asthma management was also shown as a decreased proportion of young men exempted from military service [3,4].

Among younger adults exacerbations requiring hospitalizations frequently reflect a failure in asthma management. At older age, however, co-existing chronic diseases are common [5,6]. Co-morbidities, underdiagnosis and undertreatment of asthma, and also in turn potential adverse events due to long-term asthma medication complicate the treatment of asthma and thus, increase the risk of an exacerbation [5-7]. Obesity, gastro-oesophageal reflux, sleep apnoea, hyperlipidemia, diabetes, and osteoporosis have been associated both to aging and asthma [7,8]. These conditions are also closely related to adverse effects of corticosteroids. Asthma has also been reported to double the risk of coronary heart disease [8,9]. Depression and anxiety are found more often in asthma patients and further, depression and anxiety are associated with poor asthma control, long-term work disability, and increase in emergency treatments [8,10-12]. Many of elderly asthma patients have been long-term smokers and developed irreversible airway obstruction [13]. Overall, elderly asthma patients have poorer self-estimated health status compared to that in the age matched general population. Inadequate asthma treatment is also more common in older than in younger age groups [7].

In the present study, we examined the factors that are associated with emergency visits in an elderly asthma population. In these events, asthma was the primary cause or the primary contributing factor of the visit. All visits due to dyspnea, airway infection, chest discomfort, or palpitation were included. We concentrated in the events that require the emergency department and thus, urgent actions to prevent a serious outcome [14].

## Methods

### Study population

Adult asthma patients who had visited a Pulmonary Clinic of a University Hospital during the years 1995–2006 were invited to a 10 year follow-up study. Recruitment strategy and diagnostic criteria have been described previously in detail [13]. A total of 1855 patients participated in the study. Patients asthma diagnosis was confirmed and a random sample the patients (N = 344) were studied in detail regarding their emergency treatment history. We did a retrospective comparative study between asthma patients with emergency visits (N=117, those who had ≥1 emergency visit during the years 1995-2005) and asthma patients without emergency visits (N=227, patients without emergency visits). The event free time was accounted for each patient starting from the diagnosis or year 1995 in the case diagnosis was made earlier and ending to the first emergency visit or at the latest year 2005 when the patients were contacted by the study personnel.

The study population was a random sample (N = 344) of the original asthma population (N = 1855). In the original cohort the proportion of women was 69%, mean age at diagnosis 44 years, and at recruitment 55 years.

### Assessment of emergency visits

Medical records from all health-care providers that had treated the patient were merged retrospectively. Referral diagnoses were used to identify all the ER visits at the emergency departments of the hospitals open 24/7. Visits in the primary health care were not included. Discharge diagnosis given by the physician of the emergency department was used to refine the diagnoses. All visits to the emergency clinics at hospitals open 24/7 due to asthma or respiratory infection were included into the analysis as the primary endpoint. All the visits due to upper or lower respiratory or cardiovascular systems (asthma exacerbation, respiratory infections, cardiac arrhythmias and palpitations, ischemic heart disease or heart failure, unspecified dyspnea, chest discomfort or pain) were considered as the asthma-related and were recognized in the analysis as the ER visit. Information on age, gender, BMI, smoking history and pack years, onset of chronic diseases and lung function parameters [13] were collected with a structured manner. ATS/ERS guidelines and national reference values were used in interpretation of spirometer results [15,16]. In addition, the last scheduled contact to health care prior the emergency visit was evaluated.

### Assessment of co-morbidities

Information on asthma related conditions (allergic rhinitis, asetylsalicylic intolerance, atopic eczema, chronic sinusitis, COPD, and nasal polyps), and other co-morbidities (cardio- or cerebrovascular disease, diabetes, psychiatric diseases, and alcohol abuse) were gathered in the medical records. ‘Asthma-COPD overlap’ diagnosis was given those patients who had first asthma, but who in addition developed persistent irreversible airway obstruction (FEV_1_/FVC < 70%) due to long-term smoking [17]. ‘Allergic rhinitis’ was diagnosed by a pulmonologist or a rhinologist and was based on IgE mediated allergy and rhinitis symptoms. ‘Asetylsalicylic intolerance’ was diagnosed by a pulmonologist and was based on positive symptom history. ‘Atopic eczema’ was diagnosed by a pulmonologist or a dermatologist according to eczema manifestation on typical skin areas. ‘Chronic sinusitis’ and ‘nasal polyposis’ was diagnosed by a rhinologist according to clinical symptoms and findings. Coronary or cerebrovascular disease, or peripheral arterial occlusive disease were categorized as ‘cardiovascular disease’. ‘Cerebrovascular disease’ (CVD) was diagnosed by a neurologist and was based on a stroke, brain haemorrhage or a transient ischemic attack. ‘Coronary disease’ included the patients who had been diagnosed with a myocardial infarct, an acute coronary syndrome, or angina pectoris by an internist. Both type I and type II diabetes were categorized as ‘diabetes’. ‘Psychiatric disorders’ included patients with a medication for psychotic disorders, depression, or anxiety. ‘Alcohol abuse’ included patients with chronic alcoholism and treatment because of alcohol related disorder.

### Assessment of self-reported information

Self-reported smoking status (never, ex-smoker, current smoker and pack years) was gathered by a questionnaire. In addition, health-related quality of life (HRQoL) was measured with an airway symptom specific questionnaire (AQ20 questionnaire) including 20 items [18-21]. “Yes” responses were scored as 1, and “no” and “not applicable” as 0. The summary score varied from 0 (=no symptoms or worries over the disease) to 20 (=full range of symptoms and worries about the disease). The summary scores were normally distributed among the participants. At recruitment all participants returned the questionnaire. Ambiguous or missing responses per question ranged between 1% and 2%. HRQoL was measured at the study recruitment and on average 3.3 (SD 2.9) years after the latest emergency visit. The patients also reported their working status (at work, on disability pension, on old-age pension).

The study approach was approved by the Coordinating Ethics Committee of the Hospital District and the permission to conduct this research was granted by the University Central Hospital. The study population had given their written informed consents for the study.

### Statistical analysis

The SPSS computer package (version 16.0) was used to compute differences in demographic and clinical variables. Continuous variables were compared using the non-parametric Mann–Whitney U –test and categorical variables using the Chi square test.

All associated clinical variables were included in the Cox proportional hazard model to determine the independent predictors for the first emergency visit. The respective estimated hazard ratios (HR) with confidence intervals (CI) of 95% are reported. Differences were considered statistically significant when the p value was less than 0.05.

**Figure 1:**
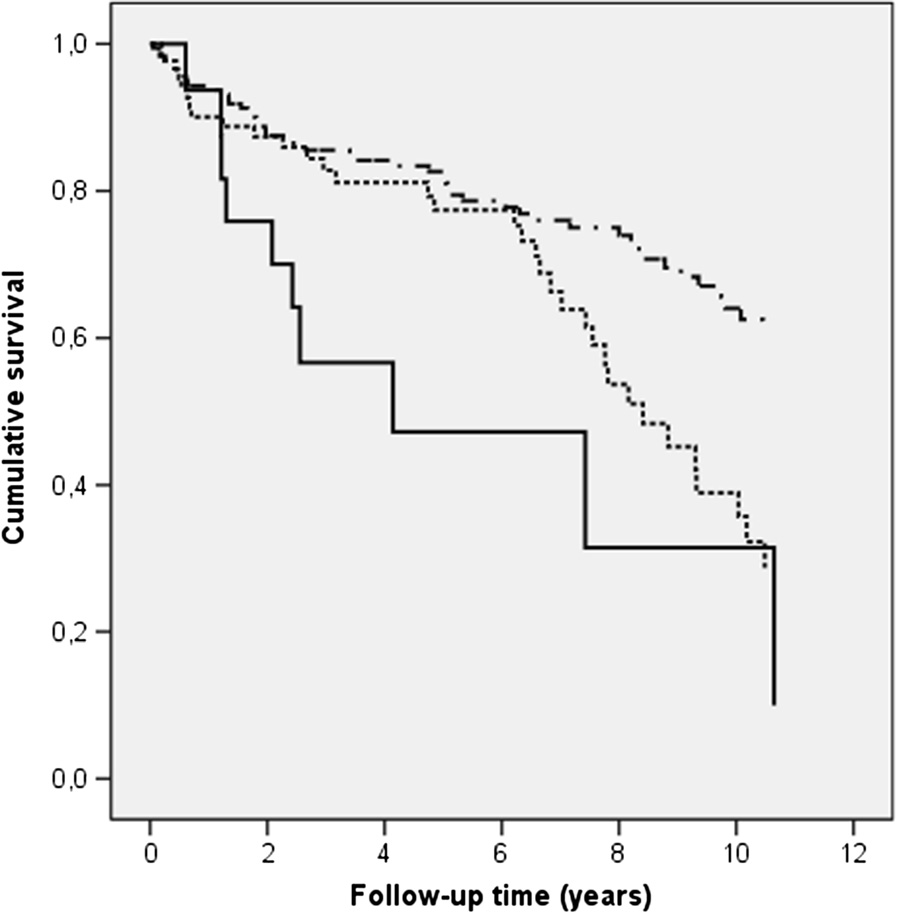
Cumulative emergency visits free time among asthma patients who were current smokers (solid line), ex-smokers (dotted line) and never smokers (dashed line) using the Cox regression model adjusted for age, gender, duration of disease, FEV1, chronic sinusitis, and number of co-morbidities.

Cumulative emergency visit free time (Figure 1) among asthma patients was stratified according to their smoking status and adjusted for age, gender, duration of disease, FEV_1_ of predicted, chronic sinusitis, and the number of co-morbidities.

## Results

### Frequency of emergency (ER) visits

A random sample (N = 344) of elderly (mean age of 56 years, 70% women) asthma patients of a Pulmonary Clinic of a University Central Hospital were included in the analysis. Their asthma related ER visits were followed through medical records since the asthma diagnosis or at the most, for 10 proceeding years (in total 1398 patient years). Altogether 117 patients (34%) had one or more (range 1–12) asthma related ER visits. These patients were considered as cases (a mean of 2.5 emergency visits per patient). Ninety-four (80%) of the cases had 1–2, and 23 (20%) had three or more asthma related ER visits. During the follow-up, the cases had in total 295 asthma-related emergency visits, a mean of 0.5 ER visits (range 0–7) per year per patient. The asthma patients with no asthma-related ER visits during the follow-up comprised the control group (N = 227).

### Differences in clinical characteristics

The patients with ER visits were on average four years older and had also suffered asthma on average four years longer than the controls (Table 1). The cases were more frequently current (14.5% vs 4.0%) or ex-smokers (38.2% vs 27.1%, p ≤ 0.001 for the trend), reported more pack years (13.2 vs 8.4, p = 0.02), displayed lower lung functions in spirometry (mean FEV1 75% vs 87% of predicted, p < 0.001), and were more frequently diagnosed with COPD second to asthma (23% vs. 8%, p < 0.001) than the controls. The mean BMI did not differ statistically significantly between the groups. A trend of an excess of co-morbidities (cardiovascular diseases, psychiatric disorders, or alcohol abuse) was found among cases compared to controls, even though not statistically significant. Except of chronic sinusitis, asthma associated allergic conditions such as allergic rhinitis, ASA intolerance, nasal polyposis, or atopic eczema did not associate with ER visits.

**Table 1:** Clinical characteristics of the asthma patients who had visited emergency clinic at least once during the follow-up compared to those who had not

	***Emergency visit***	***Emergency visit***	
***Clinical characteristics***	***Yes***	***No***	***p-value***^***1***^
**N of patients**	117	227	
**Proportion of women**	65.8%	75.3%	0.06
**Age at diagnosis** (mean ± SD)	42.6 ± 16.2	43.7 ± 17.1	not significant
**Age at recruitment** (mean ± SD)	58.5 ± 11.8	54.7 ± 12.6	0.006
**Duration of asthma, years** (mean ± SD)	15.7 ± 14.3	11.1 ± 12.0	0.001
**BMI** (mean ± SD)	26.8 ± 5.5	26.5 ± 5.6	not significant
**Smoking status** (%)			
**never**	47.3%	68.9%	0.001(trend)
**ex smoker**	38.2%	27.1%	
**current smoker**	14.5%	4.0%	
**Pack years** (mean ± SD)	13.2 ± 19.7	8.4 ± 17.4	0.02
**COPD as second diagnosis** (%)	23.1%	8.4%	<0.001
**Baseline FEV1 percentage of expected** (mean ± SD)	75.3 ± 21.1	86.6 ± 17.2	<0.001
**Baseline FVC percentage of expected** (mean ± SD)	82.9 ± 17.2	91.3 ± 14.6	<0.001
**Baseline PEF percentage of expected** (mean ± SD)	80.7 ± 20.3	94.0 ± 19.6	<0.001
**Baseline FEV1/FVC ratio** (mean ± SD)	73.4 ± 11.8	77.9 ± 10.5	0.001
**HRQoL score** (mean ± SD)	8.9 ± 4.7	6.3 ± 4.5	<0.001
**Proportion (%)of patients with**			
**no co-morbidities**	59.8	71.8	0.06
**1 co-morbidity**	33.3	23.3	
**≥2 co-morbidities**	6.8	4.8	
**Proportion (%) of patients with co-existing**			
**Cardiovascular disease**^**2**^	16.2	9.3	0.06
**Psychiatric disorder**^**3**^	21.4	16.3	not significant
**Diabetes**	3.4	5.7	not significant
**Alcohol abuse**	7.0	4.0	not significant
**Chronic sinusitis**	16.1	8.5	0.04
**Allergic rhinitis**	39.3	35.1	not significant
**Allergic exzema**	19.3	20.9	not significant
**ASA intolerance**	10.4	6.6	not significant
**Nasal polyposis**	10.3	8.0	not significant

^1^Continuous variables were compared using nonparametric Mann–Whitney U-test and categorical variables using the Chi-Square test.

^2^Included coronary disease, cerebrovascular disease, and peripheral arterial occlusive disease.

^3^Medication used.

In the end of the retrospective follow-up, the cases reported poorer respiratory specific HRQoL than the controls (total score 8.9 vs. 6.3, p = 0.001). Further, those with ≥3 ER visits had lower HRQoL than those with 1–2 visits (total score mean 11.2 vs. 8.3, p = 0.003) and their PEF values were lower than that among the controls (71.9% vs. 82.9% of the predicted, p < 0.0.001).

There were no statistically significant differences in work status between the cases and controls. Among the cases 39% were in active workforce, 35% were on old-age, and 21% on disability pension. Among the controls, the corresponding proportions were 45%, 28%, and 14%, respectively.

When the participants’ use of health care services were analysed prior to the ER visit, the last scheduled visit due to asthma was made an average 115 days (range 1–1588) prior. The last visit due to asthma was made in 64% in specialist care, and in 36% in primary health care, communal or occupational health care or at private doctor's office (Table 2). In 93% of the emergency visits, the last control had occurred within one year prior to the ER visit and no statistically significant difference was observed between primary health care and specialist care among those whose latest control exceeded one year (Table 2).

**Table 2:** Last scheduled, asthma-related visit in primary health care or in specialist care before the ER visit

	***Last scheduled visit before the ER visit***		
	***<1 year prior N = 250 (%)***	***≥1 year prior N = 19 (%)***	***TotalN = 269 (%)***
Primary health care	88 (35.2)	8 (42.1)	96 (35.7)
Specialist care	162 (64.8)	11 (57.9)	173 (64.3)
Total	250 (100)	19 (100)	269 (100)

### Reasons for ER visits among elderly asthma patients

The reason for the ER visits were analysed by using both the referral information and the more refined discharge diagnosis data. According to the both referral and the discharge diagnoses, the majority of emergency visits (47.4% and 44.6%, respectively) were due asthma exacerbation (Table 3). Asthma exacerbation was also the most common reason in hospitalizations (N = 168, 57% of all the visits). 22.4% of the visits were referred to emergency room care with a suspicion of respiratory infection, sinusitis or fever. The proportion of infections did not change in the ER evaluation. 14.1% of the ER visits was estimated to be of cardiovascular origin (pulmonary embolism, arrhythmia, ischemic coronary disease, or heart failure). 13% of the visits remained unspecified (dyspnoea, thoracic pain, hyperventilation, haemoptysis) when the patient was discharged from the emergency department.

**Table 3:** Referral and discharge diagnoses of the emergency visits made by the asthma patients

***Diagnosis***	***Reference visit N = 117 (%)***	***All visits N = 295(%)***	***Visits leading to hospitalization N = 168 (%)***
***Referral***			
**Asthma exacerbation**	50 (42.7)	140 (47.4)	87 (51.8)
**Arrhythmias**	5 (4.3)	22 (7.5)	4 (2.4)
**Chest pain**	15 (12.8)	25 (8.5)	11 (6.5)
**Dyspnoea**	19 (16.2)	40 (13.5)	26 (15.5)
**Fever**	5 (4.3)	26 (8.8)	15 (8.9)
**Respiratory infection**	17 (14.6)	33 (11.2)	22 (13.1)
**Sinusitis**	5 (4.3)	7 (2.4)	2 (1.2)
**Other causes**	1 (0.9)	2 (0.7)	1 (0.6)
**Total**	117 (100)	295 (100)	168 (100)
***Emergency discharge***			
**Asthma exacerbation**	50 (42.7 )	135 (44.6 )	86 (51.2)
**Asthma-COPD overlap**	6 (5.1)	8 (2.9)	5 (3.0)
**Pneumonia**	11 (9.4)	22 (8.7)	21 (12.5)
**Unspecified respiratory infection**	9 (7.7)	26 (8.6)	12 (7.1)
**Otitis**	1 (0.9)	6 (2.2)	2 (1.2)
**Sinusitis**	4 (3.4)	12 (4.2)	3 (1.8)
**Dyspnoea**	3 (2.6)	4 (1.9)	2 (1.2)
**Pulmonary embolism**	3 (2.6)	4 (1.3)	4 (2.4)
**Arrhythmia**	4 (3.4)	20 (6.4)	3 (1.8)
**Coronary disease or suspicion**	9 (7.7)	15 (4.8)	10 (5.9)
**Heart failure**	1 (0.9)	4 (1.6)	4 (2.4)
**Thoracic pain**	8 (6.8)	11 (3.8)	2 (1.2)
**Allergic reaction**	1 (0.9)	4 (1.3)	1 (0.6)
**Hyperventilation**	4 (3.4)	6 (2.2)	2 (1.2)
**Haemoptysis**	1 (0.9)	2 (0.6)	1 (0.6)
**Other causes**	2 (1.8)	16 (5.4)	10 (6.0)
**Total**	117 (100)	295 (100)	168 (100)

The role of clinical determinants in the occurrence of ER visits was estimated by using the Cox-regression analysis. Previous (HR 1.8) and current smoking (HR 3.9) compared to never smoking, impaired lung functions (FEV_1_ < 65%, HR 2.9) compared to normal lung function, secondary diagnosis of COPD (HR 2.3), and poor HRQoL (HR 1.4) increased the risk significantly (Table 4). Current smoking (HR 3.6), poor HRQoL (HR 2.5), and FEV_1_ < 65% (HR 2.2) remained independent risk factors for ER visit when the model was adjusted for gender, duration of asthma, cardiovascular disease, chronic sinusitis, and COPD. The ER visit free time was significantly shorter among current smokers compared to that among the ex- and never smokers (Figure 1).

**Table 4:** Hazard ratios and their 95% confidence intervals (CI) for patient characteristics that explained emergency visits among the asthma patients by the Cox regression analyses

	***N of patiens***^***1***^	***% of patients with emergency visits***	***Crude HR***	***95% CI***	***P-value***	***Adjusted HR***	***95% CI***	***P-value***
**Male gender**	96	34.2%						
**Female gender**	248	65.8%	0.68	0.46-1.00	0.051	1.11	0.67-1.84	NS
**Age until first emergency care visit or at evaluation (increase of 1 year)**			1.02	1.00-1.03	0.051	1.01	0.99-1.03	NS
**Duration of asthma**								
**<15 years**	231	60.0%	ref. 1.00			ref. 1.00		
**>=15 years**	100	40.0%	1.06	0.72-1.56	NS	1.29	0.82-2.03	NS
**FEV1% of expected**								
**> 80%**	164	42.7%	ref. 1.00			ref. 1.00		
**FEV1 65-80%**	65	24.5%	**1.90**	**1.16-3.11**	**0.011**	1.57	0.91-2.71	NS
**FEV1 < 65%**	54	32.7%	**2.90**	**1.85-4.54**	**<0.001**	**2.20**	**1.29-3.74**	**0.004**
**Smoking**								
**Never**	174	47.3%	ref. 1.00			ref. 1.00		
**Ex-smoker**	90	38.2%	**1.75**	**1.14-2.66**	**0.10**	**1.86**	**1.30-3.06**	**0.015**
**Current**	23	14.5%	**3.94**	**2.20-7.07**	**<0.001**	**3.59**	**1.58-8.15**	**0.002**
**AQ_20**								
**score 12 or less**	290	74.4%	ref. 1.00			ref 1.00		
**score 13 or more**	54	25.6%	**2.17**	**1.41-3.33**	**<0.001**	**2.51**	**1.52-4.15**	**<0.001**
**CVD**								
**No**	304	83.8%	ref. 1.00			ref. 1.00		
**Yes**	40	16.2%	1.47	0.89-2.44	NS	1.61	0.91-2.91	NS
**Sinusitis**								
**No**	299	83.9%	ref. 1.00			ref 1.00		
**Yes**	47	16.1%	1.47	0.87-2.51	NS	1.10	0.60-2.02	NS
**COPD as a second diagnosis**								
**No**	298	76.9%	ref. 1.00			ref 1.00		
**Yes**	48	23.1%	**2.32**	**1.45-3.71**	**<0.001**	0.60	0.29-1.23	NS

## Discussion

In this hospital-based adult asthma study population, one third of the patients had had an asthma related ER visit (mean 0.5 visits per patient per year). Asthma exacerbation and upper or lower respiratory tract infections covered 72% of the ER visits. Rest of the visits were due to other respiratory (15%) or cardiovascular events (13%). 57% of the ER visits led to hospitalization of the patient. The patients with emergency visits had experienced asthma longer (on average 16 years at the recruitment) than the control group (on average 11 years), and their asthma was more severe according to the lung function test results (FEV_1_ 75% vs. 87% of the predicted, respectively). 59% of the patients with ER visits had been in special care prior to the ER visit also reflecting the severity of asthma. Asthma patients with ER visits were more frequently ex- or current smokers and displayed asthma/ COPD overlapping features. Poor airway specific HRQoL was associated with the number of ER visits. Current smoking, poor HRQoL, and moderate to severe airway obstruction (FEV1 less than 65%) remained independent risk factors for ER visits in the cohort.

Since the availability of health care services has an impact on ER visits and our study design was completely based on retrospective data, the analysis was conducted only in one hospital district. The number of study subjects was small which decreased variability between the patients. Also, the small recruitment rate was limited and it was not possible to analyse further the non-responders. The follow-up time, however, was 1398 patient years, which increased the informativeness of the study design and the robustness of the major findings. When frequent visitors of ER have been studied in US, several of them had low income, inadequate medication, and no written asthma action plan [22]. By the implementation of the National Asthma Program special attention was paid to improve patients’ capabilities to self-treatment and to the health care organisations to avoid simple caps in the asthma treatment. More than 90% of the ER visitors had had a schedule appointment due to asthma within a year and 57% of all visits led to hospital admissions suggesting rather well organised asthma care in the region.

Asthma and asthma related co-morbidities have shown to be important determinants of acute care. Co-morbidities have been associated in 9% of all ER room visits and 6% of all hospitalisations in asthma [23]. Our study supports the role of co-morbidities and expands findings on smoking and the level of bronchus obstruction as risk factors of emergency care. Current smoking already in mild asthma has been associated with impaired response to inhaled corticosteroids [24-26]. With the same dose these patients report poorer asthma control including more frequent symptoms and lower peak flow values than their non-smoking counterparts. Furthermore, smoking cessation has been reported to gain better lung function in asthmatics compared to those asthma patients who continue to smoke [24-26]. Differential diagnostics with other conditions and overlap with COPD has been previously reported as a challenge for diagnosis of asthma, while co-morbidities being more of a challenge for asthma management [5].

The importance of obstruction was shown also in a prospective study of 554 asthma patients (18-55 years of age) identified from a large health maintenance organization. The patients were followed for 30 months to identify risks for acute care. The strongest predictors were airflow obstruction (RR 4.3 when FEV1<60%) and again, current smoking (RR 1.6) [27]. Typically spirometer measurements are performed when the patient is at his best, not during the exacerbations. Thus most likely declined spirometer values reflected permanent loss of lung function among this patient group. Whether the ER visit resulted due to severe disease or insufficient medication can not be determined without detailed information on used asthma medication. Other risk factors were regular exposure to solvents and the ownership of dog or cat when being skin-prick test positive. In the present older and hospital based patient cohort, allergic rhinitis or atopic eczema did not did not associate with ER visits or hospitalizations.

Allergen sensitation and exposure with simultaneous viral infection have been reported to increase the risk (OR 8.4) of hospital admissions among adult asthma patients [28]. In the present study chronic sinusitis increased the risk and acute infections explained one fourth of all visits. Tobacco smoke has been shown to increase viral replication [29,30] while inhaled corticosteroids protect against rhinovirus induced airway inflammation but not against viral replication which might partly explain poor treatment response among smokers [31]. The cases had more common co-morbidities, but compared to infections for example the significance of cardiovascular disease was much less, 13% of the visits. Diabetes, psychiatric disorders, and alcohol abuse, even though common risk factors of poorer control of asthma and acute care, were not increased in the ER-group (Table 1). Many co-morbidities such as anxiety and depression also have an impact on HRQoL and poor HRQoL on the other hand is a risk factor for ER visits [22,32,33]. Poor HRQoL also associates with known asthma triggers such as respiratory infections, environmental irritants, emotions, allergens, weather, and exercise. The patients who need acute care have frequently multiple triggers [34].

Recently, increase in severity of asthma has been suggested to be associated with an increase of obesity [35]. Obesity is associated with gastroesophageal reflux disease, hyperlipidemia, and diabetes with may in turn worsen the asthma symptoms [9]. In the study of Mosen et al. obese individuals with persistent asthma reported more frequently poorer asthma-related quality of life and more asthma-related hospitalisations than patients with normal weight [36]. Obesity remained an independent risk for hospitalizations after adjustment for age, sex, smoking status, use of corticosteroids, and gastroesophageal reflux disease. In present cohort, the BMI did not differ between the cases and controls which may be partly due to the relatively small study size.

## Conclusions

The majority of the ER visits in adult asthma were directly due to asthma exacerbation or respiratory infections indicating the importance of the education of the patients. They should have the means to recognise the symptoms early on and adapt their self-management plan accordingly. Smoking cessation plays the key role in the maintenance of normal pulmonary function and prevention of emergency care.

## Competing interests

The authors declare that they have no competing interests.

## Authors’ contributions

PK participated in the design and analysis of the study, and drafting and revising of the manuscript. HK participated in the analyses of the study and revising of the manuscript. AK participated in the acquisition of data and revising of the manuscript. TH participated in the design of the study and revising of the manuscript. TL participated in the acquisition of data, design of the study, and revising of the manuscript. All the authors have approved the final version to be published.

## Pre-publication history

The pre-publication history for this paper can be accessed here:

http://www.biomedcentral.com/1471-2466/14/119/prepub
